# Laparoscopic Hysterectomy for Uterine Adenomyosis in Patients With a History of Renal Transplant: A Case Report and Review of Literature

**DOI:** 10.7759/cureus.39410

**Published:** 2023-05-23

**Authors:** Takuro Higashi, Shinichi Togami, Yuriko Higashi, Akio Tokudome, Hiroaki Kobayashi

**Affiliations:** 1 Department of Obstetrics and Gynecology, Kagoshima University, Kagoshima, JPN; 2 Department of Obstetrics and Gynecology, Faculty of Medicine, Kagoshima University, Kagoshima, JPN; 3 Department of Obstetrics and Gynecology, Kagoshima University Hospital, Kagoshima, JPN

**Keywords:** uterine myoma, three-dimensional computed tomography, uterine adenomyosis, renal transplant, laparoscopic hysterectomy

## Abstract

Renal transplantation is a viable treatment option for patients with end-stage kidney disease; however, it requires careful surgical manipulation as the transplanted kidney is placed in the iliac fossa. Herein, we report a case of a 41-year-old female with a history of two renal transplants who presented with hypermenorrhea and dysmenorrhea. Computed tomography revealed transplanted kidneys in the bilateral iliac fossae (right atrophic), and magnetic resonance imaging showed uterine adenomyosis. Three-dimensional computed tomography was performed to determine the relationship between the arteriovenous vessels, iliac vessels, and ureter of the transplanted left kidney. A diamond-shaped trocar was inserted while monitoring the transplanted kidney. Total laparoscopic hysterectomy and bilateral salpingectomy were performed without any perioperative complications. Immunosuppressants were continued postoperatively. Laparoscopic surgery for gynecological diseases can be advantageous and should be considered in patients who underwent renal transplants.

## Introduction

Renal transplantation is considered the most effective treatment option for patients with end-stage renal disease owing to advancements in surgical techniques and immunosuppression [1-3].

As the transplanted kidney is usually placed in the iliac fossa, pelvic surgical manipulation must be performed carefully. Thus, a total hysterectomy is performed via open surgery [4-6]. Total laparoscopic hysterectomy (TLH) has been increasingly performed to treat benign uterine diseases; however, few reports have documented its application among patients who underwent renal transplantation [7,8]. We present a case of TLH in a patient who underwent renal transplantation and developed uterine adenomyosis.

## Case presentation

A 41-year-old female (gravida 1, para 1) with a history of cesarean section presented to our outpatient department for the treatment of uterine adenomyosis. She had undergone renal transplantations at the ages of 24 and 30 owing to chronic renal disease caused by immunoglobulin A nephropathy. Her father and sister were the first (right kidney) and second (left kidney) living kidney donors, respectively. She underwent a second transplant because the function of the previously transplanted kidney deteriorated. Following the second transplant, the patient’s renal function was preserved by administering immunosuppressants and antihypertensives. Two years prior, she experienced severe dysmenorrhea and hypermenorrhea and was diagnosed with uterine adenomyosis at her previous hospital. Conservative treatment failed to improve her symptoms, and she was referred to our department for surgery. Initial physical examination revealed a 15-cm surgical wound from the renal transplantations in the lateral aspect of the bilateral lower abdomen. Additionally, a 10-cm cesarean section wound was noted in the midline of the lower abdomen. The patient had an enlarged uterus but normal-sized ovaries. Transvaginal ultrasonography revealed myometrial thickening and uterine adenomyosis was diagnosed.

**Figure 1:**
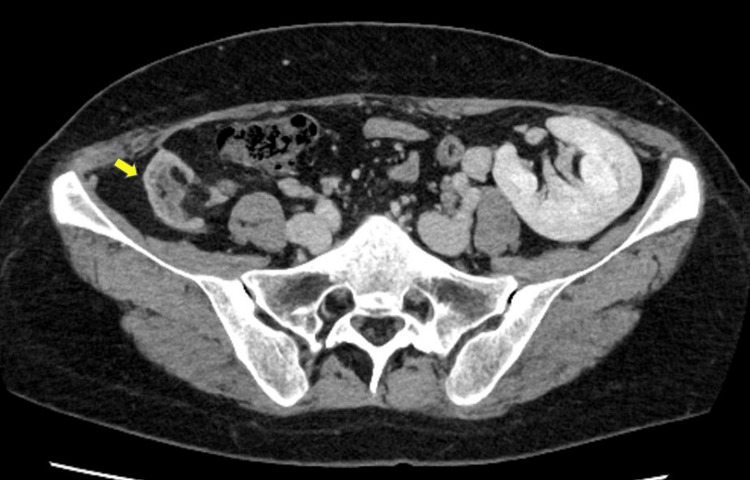
Computed tomography with contrast Computed tomography with contrast shows the bilateral transplanted kidneys in the iliac fossae. The right transplanted kidney is atrophic (arrow).

Cervical and endometrial cytology were performed, and no malignant findings were observed. The patient had a normal preoperative serum creatinine level (0.74 mg/dL), estimated glomerular filtration rate (69.1 mL/min/1.73 m^2^), and serum cancer antigen 125 level. Magnetic resonance imaging showed an enlarged uterus, thickened myometrium, and adenomyosis. The bilateral ovaries were normal. Computed tomography (CT) with contrast showed bilateral transplanted kidneys in the iliac fossae and an atrophic right kidney (Figure 1)

**Figure 2:**
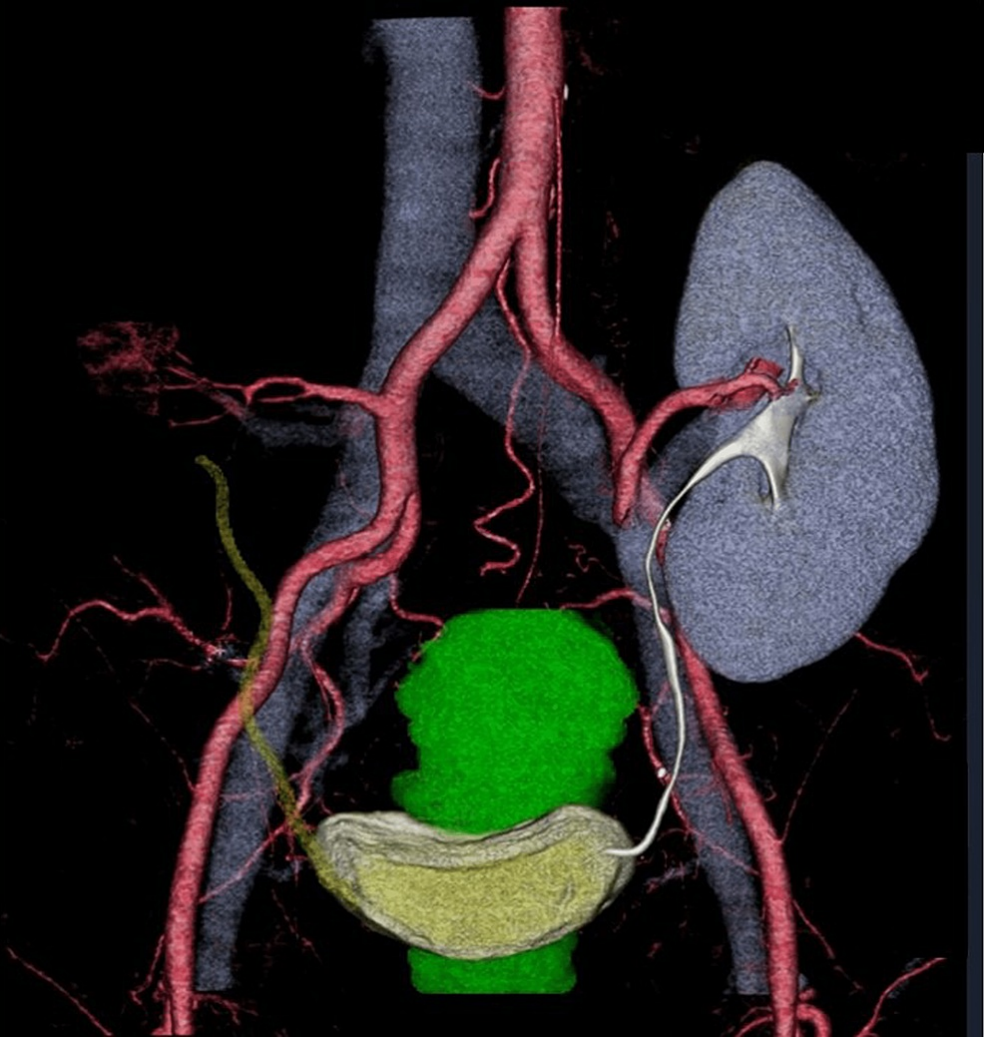
Three-dimensional computed tomography Three-dimensional computed tomography reveals the relationship between the arteriovenous vessels of the transplanted left kidney and left iliac vessels and the left ureter of the transplanted left kidney.

Additionally, a three-dimensional CT was performed to determine the relationship between the arteriovenous vessels, iliac vessels, and ureter of the transplanted left kidney (Figure 2).

**Figure 3:**
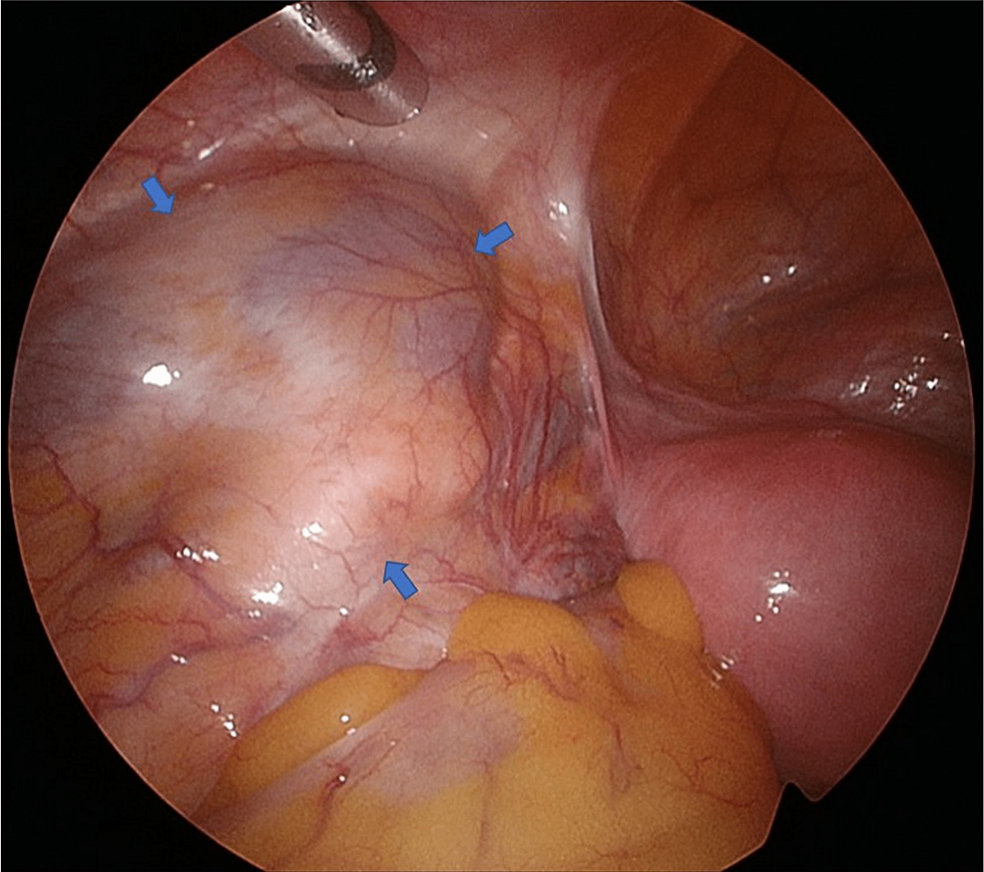
The transplanted left kidney The transplanted left kidney is present in the left iliac fossa (arrow).

The patient was hospitalized one day before surgery. She underwent TLH and bilateral salpingectomy for uterine adenomyosis 18 months after the initial visit. Immunosuppressive and antihypertensive medications were administered on the morning of the surgery. General anesthesia was administered without epidural anesthesia. First, a 5-mm camera port was inserted in the umbilicus, and four ports were inserted to form a diamond shape. The transplanted kidneys were visible in the bilateral iliac fossae (right atrophic) (Figure 3); the retroperitoneal cavity was difficult to expand owing to adhesions.

Generally, the uterine artery is isolated and ligated at its origin; however, given the risk of ureteral and vascular injury, isolation and ligation were not performed. Other surgical operations were feasible. The operative time was 72 min, and the estimated blood loss was 5 mL. No intra/postoperative complications occurred. Postoperative renal dysfunction was not observed. Immunosuppressants and antihypertensives were administered one day postoperatively without drug withdrawal. The patient started walking and eating on postoperative day 1 and was discharged on postoperative day 5.

## Discussion

We report a case of uterine adenomyosis in a 41-year-old female patient who underwent TLH after renal transplant surgery. In renal transplants, the transplanted kidney is placed in the iliac fossa, and the renal vessels are anastomosed with the external or internal iliac vessels. Additionally, if the transplanted kidney is larger than the original, the trocar should be inserted carefully to avoid damaging the transplanted kidney. Here, a camera port was inserted through the umbilicus, and the remaining trocars were safely inserted while confirming the location of the transplanted kidney using a camera. In previous reports, transabdominal echocardiography has been used to verify the location of the transplanted kidney during insertion [8]. The ureter from the transplanted kidney is anastomosed with the bladder. The course of the ureter may vary and may not be appreciated in the abdominal cavity. Moreover, the retroperitoneum expands during transplantation surgery, and adhesion formation is likely. In TLH, the uterine artery is isolated and ligated at its origin; however, given the risk of ureteral and vascular injury in this case, isolation and ligation were not performed. The left Okabayashi para-rectal space was unfolded, and the vessels and ureters of the transplanted and original kidneys were dissected externally to process the parametrium safely. Furthermore, the blood vessels and ureters of the transplanted kidney were localized using three-dimensional CT before surgery to ensure safety.

In patients who underwent renal transplantation, a hysterectomy is performed via open surgery to address gynecological disorders. In a previous report of 42 patients who underwent open total hysterectomy after renal transplantation, 24 (41.4%) developed complications, with the most common being infection (n = 15) and blood transfusion (n = 8) [6]. Adequate perioperative fluid, electrolyte correction, and continued oral immunosuppression are essential in managing postrenal transplant patients. However, perioperative complications can complicate postoperative patient management. Laparoscopic hysterectomy is a viable treatment option for gynecological diseases and should be considered for patients with a history of renal transplantation owing to its advantages. First, the procedure results in a small wound, with minimal risk of postoperative wound infection. Second, the postoperative pain is minimal; therefore, the need for analgesics is reduced. Third, hospitalization duration and risk for nosocomial infection are decreased. Finally, the patient can rapidly return to oral feeding, and the risk of bowel obstruction is reduced, allowing the continued use of immunosuppressants [7,9]. Here, the patient had mild postoperative wound pain, was discharged without any perioperative complications, and immunosuppressive drugs were administered without withdrawal.

Patients who undergo laparoscopic surgery have been shown to experience decreased urinary output owing to reduced renal blood flow caused by pneumoperitoneal pressure. When the pneumoperitoneal pressure exceeds 15 mmHg, the increased renal venous pressure could result in oliguria [10]. Therefore, the effect of pneumoperitoneal pressure on renal function should be monitored intraoperatively. Here, pneumoperitoneum was induced at 8 mmHg for 63 min; however, the intraoperative urine output was maintained at approximately 2.8 mL/kg/h.

## Conclusions

With the growing number of renal transplantations, the number of laparoscopic surgeries to address gynecological diseases in patients with previous renal transplantations is expected to increase. The perioperative management of patients with a history of renal transplantation consists of surgical techniques that take into consideration the anatomical changes caused by renal transplantation, as well as the renal dysfunction and infections caused by immunosuppression.
